# Risk of dementia associated with cardiometabolic abnormalities and depressive symptoms: a longitudinal cohort study using the English longitudinal study of ageing

**DOI:** 10.1002/gps.5019

**Published:** 2018-11-27

**Authors:** Panagiota Kontari, Kimberley J. Smith

**Affiliations:** ^1^ Department of Psychological Sciences, School of Psychology, Faculty of Health and Medicine University of Surrey Guildford UK

**Keywords:** cardiometabolic risk factors, dementia, depression

## Abstract

**Objectives:**

Depression and cardiometabolic abnormalities are independently associated with a high risk of dementia. This study aimed to examine the association of comorbid depressive symptoms and cardiometabolic abnormalities with risk of dementia.

**Methods:**

The sample comprised 4859 participants aged 50 or older without baseline dementia who took part in the English Longitudinal Study of Ageing (waves 2‐7). Depressive symptoms were assessed using the Center for Epidemiologic Studies‐Depression tool. Cardiometabolic abnormalities were defined as three or more cardiometabolic risk factors (inflammation, central obesity, raised triglycerides, low high‐density lipoprotein [HDL] cholesterol, hypertension, and hyperglycaemia or diabetes). Participants were classified into four groups based on presence of depressive symptoms and cardiometabolic abnormalities. Results were analysed using the Cox proportional hazards regression adjusted for covariates.

**Results:**

A total of 216 cases of incident dementia were reported over 10 years of follow‐up. The group with high depressive symptoms only had an increased hazard of developing incident dementia during follow‐up (HR = 2.68; 95%CI, 1.70‐4.23), which was attenuated after adjustment for baseline cognition. No evidence was found for an association of overall cardiometabolic abnormalities with incident dementia; though hyperglycaemia, hypertension, and abdominal obesity with depressive symptoms had an unadjusted association with incident dementia. Only low‐HDL cholesterol with depressive symptoms had an adjusted association with incident dementia (HR = 0.18; 95%CI, 0.04‐0.75).

**Conclusions:**

This work confirms depressive symptoms as a risk factor for incident dementia. However, low HDL‐cholesterol with depressive symptoms may be protective against dementia, though more work is required to confirm this association.

**Key points**

- High depressive symptoms without cardiometabolic abnormalities are associated with an increased hazard of dementia.
- Cardiometabolic abnormalities (with or without depressive symptoms) are not associated with an increased hazard of dementia.
- Individual cardiometabolic abnormalities with depressive symptoms (such as hyperglycemia, hypertension, and abdominal obesity) are associated with an increased unadjusted hazard of dementia; though these associations are attenuated after adjustment.
- After adjusting for all confounders low HDL‐cholesterol with depressive symptoms are predictive of a decreased risk of developing dementia.

## 1 INTRODUCTION

Dementia is a neurodegenerative disorder of later life characterised by progressive deterioration of cognitive function, beyond what might be expected from normal ageing.^1^ As our population ages, it is projected that the number of people affected by dementia will increase.^2^

There is currently no cure and neuropathological mechanisms, underlying dementia, take place over many years before the overt dementia symptoms emerge.^3^

Recently, great attention has been paid to the effect of cardiometabolic abnormalities as a potential trigger of dementia.^4^, ^5^ Cardiometabolic abnormalities include cardiovascular risk factors such as central obesity, raised triglycerides, low high‐density lipoprotein (HDL) cholesterol, hypertension, high blood glucose, and increased inflammation.^6^, ^7^

However, the evidence‐base regarding the association of cardiometabolic abnormalities with cognitive decline and incident dementia is mixed.^8^, ^9^, ^10^, ^11^, ^12^ Some studies indicate cardiometabolic abnormalities increase the risk of dementia,^9^, ^12^ whereas others find no association.^10^, ^11^ Some researchers, instead, propose that individual markers of cardiometabolic abnormalities are linked with dementia rather than combined cardiometabolic abnormalities.^11^ Given that there are mixed findings about the relationship of each cardiometabolic factor and their additive impact on risk of dementia, more research is needed to determine this potential link.

Cardiometabolic abnormalities have been shown to be strongly linked with depression,^13^ which is also an independent risk factor for incident dementia.^14^, ^15^ Previous work suggests that the co‐occurrence of cardiometabolic abnormalities and depression might represent a subtype of depression called “metabolic depression.”^16^, ^17^ This comorbidity is associated with lifestyle and health‐related risk factors (eg, poor self‐care) for cardiovascular and metabolic diseases.^18^, ^19^ These cardiovascular and metabolic diseases have been shown to be risk factors for development of future dementia.^20^ Furthermore, work shows that co‐occurring cardiometabolic abnormalities and depressive symptoms are risk factors for cognitive decline.^21^

However, to the best of our knowledge, no previous work has examined whether co‐occurring cardiometabolic abnormalities and depression might be associated with incident dementia. The aim of the present study was to examine the impact of elevated depressive symptoms and cardiometabolic abnormalities on the incidence of dementia in a large community‐dwelling sample of adults aged 50 years and older for more than 10 years.

## 2 METHODS

### 2.1 Participants

The sample of this study was recruited from the English Longitudinal Study of Ageing (ELSA), a prospective cohort study of community‐dwelling individuals aged 50 and older in England. ELSA consists of men and women, born before 1 March 1952, who took part in the Health Survey for England (HSE) from 1998 to 2001 and lived in a private house in the first wave of study.^22^

For this study, data from wave 2 (2004‐2005) were used as baseline, as it was the first wave to involve clinical assessments and collection of blood samples by research nurses. Data from waves 3 (2006‐2007) to 7 (2014‐2015) were used as the follow‐up phases for identifying newly diagnosed dementia. The analytical sample involved participants with complete data on depressive symptoms, with valid blood sample, and no more than two missing values for the six cardiometabolic risk factors assessed. The study restricted the analyses to participants who attended at least one subsequent follow‐up session (wave 3‐7) after the baseline assessment (wave 2). Participants with dementia or Alzheimer's disease (AD) at or before the baseline assessment were excluded.

All participants provided signed full informed consent, and ethical approval for all the ELSA waves was granted by the London multicentre research ethics committee.

## 3 MEASURES

### 3.1 Outcome

#### 3.1.1 Dementia incidence

The outcome variable was defined as time‐to‐dementia from the index date identified as the date of the wave 2 interview. The incidence of dementia was determined during the follow‐up period between waves 3 to 7. This was determined in one of two ways. Firstly, the identification of newly diagnosed dementia based on participant or informant reported physician‐diagnosed dementia or AD. Date to time of event was determined by self‐report, or where date of diagnosis was not provided. This was calculated as the midpoint between the previous interview and current interview in line with previous work.^20^ Secondly, incidence of dementia determined with the 16‐item Informant Questionnaire on Cognitive Decline in the Elderly (IQCODE). The shortened version of IQCODE has been validated as a sensitive tool in the screening of dementia^23^ and scores performance on various cognitive, executive, and daily functions compared with the previous 10 years. The validated cut‐off point of 3.5 was used to define dementia^24^ with the date of time of event being defined as the interview date (month and year).

Those people who did not develop dementia during the follow‐up or before they were lost to follow‐up were treated as censored. The study did not distinguish between the reasons for losses to follow‐up (eg, due to death).

### 3.2 Predictors

#### 3.2.1 Depressive symptoms

The 8‐item Center for Epidemiologic Studies‐Depression (CES‐D) tool was used to evaluate self‐reported depressive symptomology. Participants were asked to respond with a binary option (yes/no) whether they had experienced different symptoms of depression, such as much of the time feeling sad during the past week. The total sum score ranged from 0 to 8. Participants were dichotomized into those with “no or low” (CES‐D < 4) and those with “high” (CES‐D ≥ 4) depressive symptomatology, in line with previous ELSA studies.^19^, ^25^

#### 3.2.2 Cardiometabolic abnormalities

Six cardiometabolic risk factors were evaluated including C‐reactive protein (CRP), central obesity, raised triglycerides, low HDL cholesterol, hypertension, and hyperglycaemia or diabetes. These risk factors were chosen as they have been used in previous work to identify cardiometabolic abnormalities.^18^, ^19^, ^25^

Central obesity was defined as having waist circumference of greater than or equal to 94 cm for European men and greater than or equal to 80 cm for European women. Raised triglycerides were greater than or equal to 1.7 mmol/L and reduced HDL cholesterol is less than 1.0 mmol/L in males and and less than 1.3 mmol/L in females. Hypertension risk was defined as having systolic blood pressure of greater than or equal to 130 mm Hg or diastolic blood pressure of greater than or equal to 85 mm Hg, or use of antihypertensive treatment. Hyperglycaemia was defined as having raised fasting plasma glucose (FPG) of greater than or equal to 5.6 mmol/L and/or having a previous diagnosis of type 2 diabetes. The cutoffs used for obesity, triglycerides, HDL‐cholesterol, hypertension, and hyperglycaemia were based on the International Diabetes Federation (IDF) criteria.^6^

Finally, systemic inflammation was determined as having CRP levels of greater than 3 mg/L, according to the American Heart Association and the Center for Disease Control.^26^

Participants were categorised as having cardiometabolic abnormalities if they exhibited a minimum of three of the above cardiometabolic risk factors in line with previous work.^19^, ^25^

### 3.3 Covariates

#### 3.3.1 Sociodemographic variables

Demographic variables involved age, gender (male/female), marital status (single/married/separated/widowed), education (higher education/high school or college/no qualifications), and nonpension net wealth (lowest to highest quintile). Ethnicity was not included since 98% of the ELSA sample were Caucasian.

#### 3.3.2 Clinical variables

Self‐reported physician‐diagnosis of cardiovascular comorbidities (angina, myocardial infarction, congestive heart failure, heart murmur, arrhythmia, and stroke) were also recorded. Reporting one or more of these conditions was defined as cardiovascular comorbidity.

#### 3.3.3 Lifestyle variables

Self‐reported health‐related behaviours included physical activity measured using the self‐reported participation in vigorous, moderate or low impact physical activities (sedentary or low/moderate/high see previous study by Smith et al^27^ for more information on categorisation), and self‐reported smoking status (never/former/current).

#### 3.3.4 Cognitive function

Cognitive function was defined as the total cognitive index derived from the total scores on memory and executive tests and ranged from 0 to 50.^22^ Specifically, memory tests included a word list learning task, which measured verbal learning, immediate and delayed memory (recall of 10 nouns), and a prospective memory task (remembering to remember). The combined scores from memory tests ranged from 0 to 27. Executive function tests measured verbal semantic fluency (naming as many animals as possible in 1 min) and attention (letter cancellation task). The combined scores from the executive function index ranged from 0 to 23.

## 4 STATISTICAL ANALYSIS

The study sample was categorised into four groups based on CES‐D scores (less than 4 low‐to‐no depressive symptomatology versus greater than or equal to 4 elevated depressive symptomatology) and cardiometabolic abnormalities (less than 3 absence of cardiometabolic risk factors versus greater than or equal to 3 presence of cardiometabolic risk factors) in line with previous studies.^19^, ^25^ The first group had no or low depressive symptoms and no cardiometabolic abnormalities. The second group had high depressive symptoms without cardiometabolic abnormalities. The third group had no or low depressive symptoms but with cardiometabolic abnormalities. The fourth group had comorbidity of high depressive symptoms and cardiometabolic abnormalities.

Cross‐tabulations (Pearson chi‐square) and one‐way analysis of variance (one‐way ANOVA) were conducted to compare the four groups on baseline sociodemographic, health, lifestyle, and cognitive variables.

Kaplan–Meier survival curves and Cox proportional hazards regressions were used to estimate the prospective associations between categories of depressive symptomatology and cardiometabolic abnormalities with dementia incidence. Cox proportional hazard models assessed the time to new events of incident dementia presented as hazard ratios (HRs) with 95% confidence intervals (95%CIs). Model 1 contained the unadjusted association between the four groups and dementia incidence. Model 2 adjusted for sociodemographic variables including age, gender, marital status, education, and net wealth. Model 3 further adjusted for lifestyle covariates and cardiovascular comorbidities. Model 4 further adjusted for cognitive function. The group with low or no depressive symptoms and no cardiometabolic abnormalities served as reference group.

Because of the observed heterogeneity in studies that examine cardiometabolic abnormalities with dementia, we conducted additional analyses where we stratified each individual by cardiometabolic indicator with depressive symptoms to determine whether there may be differences based on looking at overall cardiometabolic abnormalities or individual components. Analyses were run both unadjusted and adjusted for all covariates.

### 4.1 Sensitivity analysis

Three sensitivity analyses were conducted in order to assess the robustness of the findings of the primary analysis. To minimise the possibility that depressive symptoms occur as a prodrome of dementia, we conducted a sensitivity analysis that repeated the above proportional hazard models, excluding incident cases of dementia during the two‐year period after baseline (wave 3). This approach to assessing depression as a prodrome has also been used in previous research.^28^ We conducted a second sensitivity analysis removing those with cardiovascular comorbidity at baseline. This analysis is based on the hypothesis that a cardiovascular comorbidity may influence the risk of developing dementia in older age.^29^

A third sensitivity analysis evaluated robustness of the findings against the reliability of dementia diagnosis by excluding cases diagnosed via the IQCODE and limiting the analysis to self‐reported physician diagnosed dementia only.

Statistical analyses were conducted using the SPSS 22.0 for Windows (IBM Corporation, Armonk, NY).

### 4.2 Results

**Figure 1:**
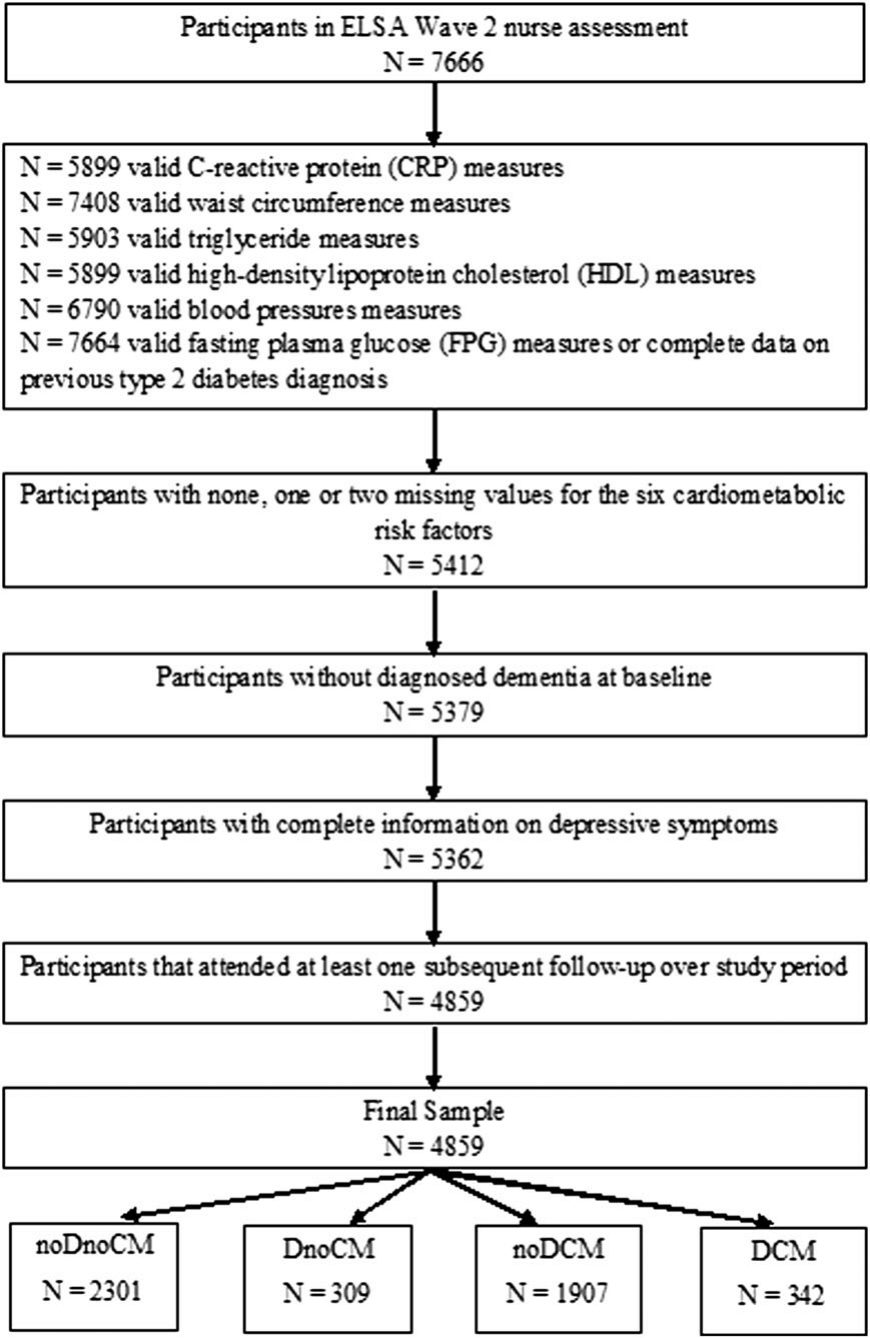
Participant flow chart. noDnoCM: no or low depressive symptoms and no cardiometabolic abnormalities group; DnoCM: high depressive symptoms only group; noDCM: cardiometabolic abnormalities only group; DCM: comorbid high depressive symptoms and cardiometabolic abnormalities group

A total of 7666 individuals participated in the nurse baseline assessment in wave 2. However, the final analytical sample comprised 4859 participants free of dementia at baseline who had sufficient data to be placed in one of the four groups (see Figure 1 for participant flowchart).

The sociodemographic, health, lifestyle, and cognitive characteristics of the analytical sample according to the four depressive symptom and cardiometabolic abnormalities groups are shown in Table 1.

**Table 1:** Sociodemographic characteristics of the participants across the depressive symptom and cardiometabolic abnormalities groups

	noDnoCM	DnoCM	noDCM	DCM	*P* value*
N (Total: 4859)	2301	309	1907	342	
Age (M ± SD)	64.87 ± 9.36	66.50 ± 11.13	66.76 ± 9.15	67.39 ± 9.37	*P* < 0.001
Gender (N, %)					
Male	1065 (46.3)	85 (27.5)	906 (47.5)	124 (36.3)	*P* < 0.001
Female	1236 (53.7)	224 (72.5)	1001 (52.5)	218 (63.7)	
Education (N, %)					
Higher education	723 (31.4)	71 (23.0)	456 (23.9)	52 (15.3)	*P* < 0.001
High school or college	935 (40.6)	117 (37.9)	732 (38.4)	111 (32.6)	
No qualification	643 (27.9)	121 (39.2)	719 (37.7)	177 (52.1)	
Net wealth (N, %)					
5 (highest)	650 (28.7)	57 (18.7)	328 (17.4)	27 (7.9)	*P* < 0.001
4	547 (24.2)	43 (14.1)	412 (21.9)	58 (17.0)	
3	454 (20.1)	67 (22.0)	418 (22.2)	68 (19.9)	
2	370 (16.3)	68 (22.3)	382 (20.3)	82 (24.0)	
1 (lowest)	243 (10.7)	70 (23.0)	342 (18.2)	107 (31.3)	
Marital status (N, %)					
Single	1626 (70.7)	144 (46.6)	1322 (69.3)	173 (50.6)	*P* < 0.001
Married	187 (8.1)	37 (12.0)	131 (6.9)	22 (6.4)	
Separate	453 (19.7)	120 (38.8)	432 (22.7)	136 (39.8)	
Widowed	35 (1.5)	8 (2.6)	22 (1.2)	11 (3.2)	
Physical activity (N, %)					
Sedentary/low	392 (17.0)	126 (40.8)	578 (30.3)	165 (48.4)	*P* < 0.001
Moderate	1280 (55.7)	139 (45.0)	1002 (52.6)	150 (44.0)	
High	628 (27.3)	44 (14.2)	326 (17.1)	26 (7.6)	
Smoking status (N, %)					
Never smoked	961 (41.8)	103 (33.4)	696 (36.5)	101 (29.5)	*P* < 0.001
Former smoker	1104 (48.0)	160 (51.9)	982 (51.5)	179 (52.3)	
Current smoker	234 (10.2)	45 (14.6)	229 (12.0)	62 (18.1)	
Cardiovascular comorbidity (N, %)					
Yes	447 (19.4)	88 (28.5)	431 (22.6)	113 (33.0)	*P* < 0.001
Cognitive function (M ± SD)	30.28 ± 6.10	28.21 ± 6.80	29.04 ± 6.01	26.67 ± 6.47	*P* < 0.001

*Note*. noDnoCM: no or low depressive symptoms and no cardiometabolic abnormalities group; DnoCM: high depressive symptoms only group; noDCM: cardiometabolic abnormalities only group; DCM: comorbid high depressive symptoms and cardiometabolic abnormalities group.

* Chi‐square for categorical variables, one‐way ANOVA for continuous variable (Age and cognitive function), and *P* values estimate the main effect among all four groups.

Notably, having both high depressive symptoms and cardiometabolic abnormalities was significantly related to being older, female, and separated. This group were less likely to have attained a higher education and be from a wealthier quintile. Participants with depressive symptoms only were also more likely to be female, less educated, and less wealthy. Participants with comorbid depression and cardiometabolic abnormalities were significantly more likely to have reported at least one cardiovascular comorbidity, as compared with other groups. As well, co‐occurrence of depression and cardiometabolic risk factors was significantly associated with lower cognitive performance compared with other three groups. Similarly, participants with high depressive symptoms only were more likely to suffer from comorbid cardiovascular disease and to have lower cognitive ability, compared with the reference and cardiometabolic abnormalities groups.

### 4.3 Incident dementia

A total of 216 (4.4%) incident cases of self‐reported physician diagnosed dementia or IQCODE score‐based diagnosed dementia were reported over an average of 96.5 months of follow‐up (wave 3‐7). Of the 216 cases, 193 were identified from self‐reports of physician‐diagnosed dementia and the remaining 23 were identified based on IQCODE score. The number of participants in each group that developed dementia of more than 10 years of follow‐up were 21 (6.1%) individuals with high depressive symptoms and cardiometabolic abnormalities, 25 (8.1%) individuals with high depressive symptoms only, 88 (4.6%) individuals with cardiometabolic abnormalities only, and 82 (3.6%) individuals without any condition at baseline.

**Figure 2:**
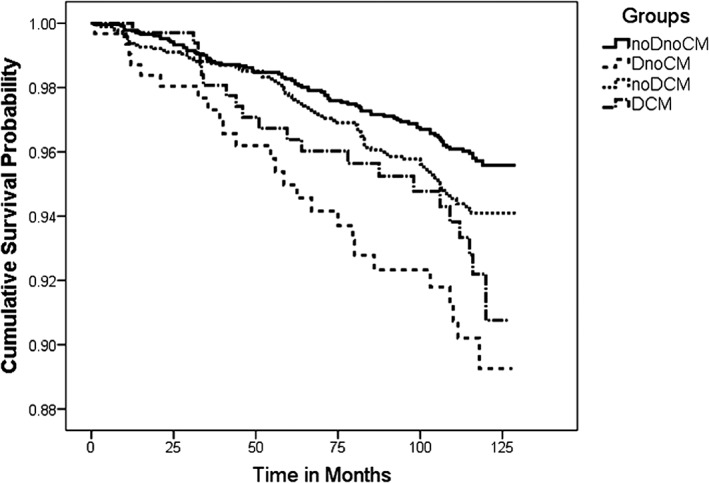
Kaplan–Meier survival curves stratified by depression and cardiometabolic abnormalities groups. The survival curves display survival probabilities (cumulative survival probability) of individuals remaining disease free at any time after baseline. The survival time was the time‐to‐dementia, measured in months. noDnoCM: no or low depressive symptoms and no cardiometabolic abnormalities group; DnoCM: high depressive symptoms only group; noDCM: cardiometabolic abnormalities only group; DCM: comorbid high depressive symptoms and cardiometabolic abnormalities group

The incidence of dementia during the study period was graphically presented using the Kaplan–Meier survival curves (see Figure 2). Participants with high depressive symptoms only exhibited the lowest cumulative survival probability in comparison with the other three groups. Those with comorbid depression and cardiometabolic abnormalities had the second lowest cumulative survival probability among the groups, whereas participants with cardiometabolic abnormalities only demonstrated slightly less probability of survival compared with the reference group. The log rank test showed that there was a statistically significant difference among the survival distributions of the different depression and cardiometabolic groups, *χ* ^*2*^(3) = 20.55, *P* < 0.001.

When assessed with the Cox proportional hazards regression high depressive symptoms without cardiometabolic abnormalities were significantly associated with an increased risk of dementia incidence in both unadjusted (HR = 2.68; 95% CI, 1.70‐4.25) and adjusted models for sociodemographic, lifestyle, and cardiovascular comorbidity covariates (HR = 1.82; 95% CI, 1.13‐2.95). Additional adjustment for cognitive function attenuated the association of elevated depressive symptoms only group with risk of dementia (HR = 1.28; 95% CI, 0.78‐2.08). No significant associations were found for the two cardiometabolic abnormalities groups for either the crude or adjusted models (see Table 2).

**Table 2:** Hazard ratio (95% CI) of dementia across depressive symptoms and cardiometabolic abnormalities groups

Cox regression HR (95% CI)	noDnoCM	DnoCM	noDCM	DCM
Model 1: Unadjusted	1.00	2.68*** (1.70‐4.25	1.32 (0.96‐1.81)	1.61 (0.95‐2.73)
Model 2: Adjusted for age, gender, education, marital status, and net wealth	1.00	2.09** (1.31‐3.36)	1.11 (0.81‐1.53)	1.08 (0.63‐1.85)
Model 3: Model 2 + adjusted for cardiovascular comorbidity, smoking status, and physical activity	1.00	1.82* (1.13‐2.95)	1.04 (0.75‐1.44	0.89 (0.51‐1.55)
Model 4: Model 3 + adjusted for cognitive function	1.00	1.28 (0.78‐2.08)	1.00 (0.73‐1.38)	0.62 (0.36‐1.09)

*Note*. HR: hazard ratio; CI: confidence interval; noDnoCM: no or low depressive symptoms and no cardiometabolic abnormalities group; DnoCM: high depressive symptoms only group; noDCM: cardiometabolic abnormalities only group; DCM: comorbid high depressive symptoms and cardiometabolic abnormalities group.

* *P* < 0.05.

** *P* < 0.01.

*** *P* < 0.001.

### 4.4 Stratification by individual cardiometabolic markers

Across all stratified analyses, high depressive symptoms alone remained a significant predictor of incident dementia. However, there were some observable differences in results based on different groupings of individual cardiometabolic abnormalities (see Table 3). Groups with both high depressive symptoms and hyperglycemia/diabetes (HR = 2.50; 95% CI, 1.31‐4.76) and high depressive symptoms with abdominal obesity (HR = 1.72; 95% CI, 1.05‐2.81) had an increased hazard of incident dementia compared with their respective reference groups (see Table 3). Furthermore, groups with either hypertension alone (HR = 1.86; 95% CI, 1.29‐2.69) or both hypertension and depressive symptoms (HR = 2.92; 95%CI, 1.79‐4.76) also had an increased hazard of incident dementia compared with a group with no hypertension and no depressive symptoms. However, these associations were attenuated after adjustment for all covariates (see Table 3). Conversely, in the fully adjusted model, low HDL‐cholesterol and depressive symptoms were associated with a decreased risk of incident dementia compared with the reference group (HR = 0.18; 95% CI, 0.04‐0.75).

**Table 3:** Hazard ratio (95% CI) of dementia across depressive symptoms and individual indicators of cardiometabolic abnormalities

Cox regression HR (95% CI)		noDnoCM	DnoCM	noDCM	DCM
Abdominal obesity	Unadjusted	1.00	3.13** (1.62‐6.03)	1.09 (0.75‐1.59)	1.72* (1.05‐2.81)

*Note*. HR: hazard ratio; CI: confidence interval; noDnoCM: no or low depressive symptoms and no indicator of cardiometabolic abnormalities; DnoCM: high depressive symptoms only group; noDCM: cardiometabolic abnormalities indicator only group; DCM: comorbid high depressive symptoms and cardiometabolic abnormalities group. To see which indicator of cardiometabolic abnormalities was assessed see column to left.

* *P* < 0.05.

** *P* < 0.01.

*** *P* < 0.001.

## 5 SENSITIVITY ANALYSES

The sensitivity analysis that excluded dementia diagnoses in the two‐year period after baseline resulted in a loss of 37 cases of dementia. In this analysis, the increased risk of dementia associated with depressive symptoms alone was slightly lower in both unadjusted (HR = 2.57; 95% CI, 1.54‐4.29) and adjusted models for sociodemographic, lifestyle, and cardiovascular comorbidity covariates (HR = 1.79; 95% CI, 1.05‐3.06). However, this association was attenuated after adjustment for cognitive function (HR = 1.57; 95% CI, 0.74‐2.19). Moreover, the group with both depressive symptoms and cardiometabolic abnormalities was significantly associated with incident dementia with only a slight change in the unadjusted model (HR = 1.82; (95% CI, 1.06‐3.16). No significant associations were found for either cardiometabolic abnormalities alone (HR = 0.98; 95% CI, 0.69‐1.40) or both cardiometabolic abnormalities and depressive symptoms (HR = 0.70; 95% CI, 0.39‐1.25) in the fully adjusted model (see Supporting Information Table A).

We performed an additional sensitivity analysis to assess the risk of dementia in those without cardiovascular comorbidity at baseline. The analysis resulted in a loss of 80 dementia cases. The groups with depressive symptoms only (HR = 2.17; 95% CI, 1.13‐4.17) and cardiometabolic abnormalities alone (HR = 1.48; 95% CI, 1.01‐2.17) showed a significant association with incident dementia in the unadjusted model, though these associations were attenuated after adjusting for confounders. No significant associations were found for the group with both depressive symptoms and cardiometabolic abnormalities for either the unadjusted or adjusted models (see Supporting Information Table B).

A third sensitivity analysis excluded cases diagnosed using the IQCODE and limited the analysis to self‐reported physician‐diagnosed dementia. This resulted in a loss of 23 events of dementia in the follow‐up period. The group with high depressive symptoms but no cardiometabolic abnormalities was the only group that was significantly associated with dementia incidence in the unadjusted model (HR = 2.14; 95% CI, 1.27‐3.58) (see Supporting Information Table C) though this association was attenuated in the fully adjusted model.

## 6 DISCUSSION

This prospective longitudinal analysis indicated that individuals with elevated depressive symptoms only had an approximately two‐fold increased risk of future dementia, and this association remained significant after adjusting for sociodemographic, lifestyle, and clinical covariates. However, neither comorbid depressive symptoms and cardiometabolic abnormalities nor cardiometabolic abnormalities alone appeared as potential risk factors for dementia over time. We did, however, find that individual indicators of cardiometabolic abnormalities with depression symptoms, specifically hyperglycaemia, hypertension, and abdominal obesity were linked with incident dementia in this population.

This study lends further support that depression may be a potential risk factor for dementia. The findings of the present study are in agreement with meta‐analyses, which demonstrate that individuals with depression are more than double at the risk of developing dementia.^14^, ^15^ In our sensitivity analysis, that excluded dementia cases in the 2 years after baseline, depressive symptoms alone were associated with a slightly lower risk of dementia than was observed in the main analysis, however it did not substantially affect estimates. Furthermore, our sensitivity analysis that excluded dementia cases diagnosed via IQCODE still showed high depressive symptomatology as a potential risk factor for developing subsequent dementia, although again, this association was only detected in unadjusted analyses. At present, the possible mechanisms behind the role of depressive symptoms in the development of dementia remain uncertain. A possible pathway linking depression with dementia could be through neurobiological changes and neuronal brain damage.^30^ Previous research suggests the relationship between depressive symptoms and dementia may also be explained in part by cardiovascular comorbidity, in which vascular damage in the brain might predispose to depression in the elderly (vascular depression hypothesis).^29^ However, our results suggest that the association between depression and incident dementia is independent of co‐occurring cardiometabolic abnormalities. Furthermore, in our sensitivity analysis, which excluded those with a cardiovascular comorbidity at baseline, depressive symptoms alone were still associated with an increased risk of dementia in unadjusted analyses (which was attenuated after adjustment). This suggests that the link between depressive symptoms and dementia may also be independent of cardiovascular comorbidity, although more work is needed to confirm this.

Prior research on the possible relationship between cardiometabolic risk factors and risk of dementia reports contradictory results.^10^, ^11^, ^12^ This discrepancy may be attributable to methodological differences and heterogeneity in the study design, population selection, criteria used for the diagnosis of dementia, thresholds used for the definition of cardiometabolic risk factors, and differences in confounder adjustment.^31^ Notably, vascular dementia (VaD), rather than AD and other dementias, is the subtype of dementia most commonly linked with cardiometabolic risk factors.^32^, ^33^ However, we were unable to assess this subtype of dementia within our study. Our research is in agreement with those studies that find no association between cardiometabolic abnormalities and risk of future dementia.^10^, ^11^, ^34^ The only significant adjusted result that we uncovered in this study regarding cardiometabolic abnormalities was that those people with low HDL‐cholesterol (indicative of hypercholesterolemia) and depressive symptoms were less likely to develop incident dementia. Some previous work has suggested that hypercholesterolemia may be protective against dementia risk.^11^, ^35^ Previous work has shown that lower blood fats are linked with an increased risk of mortality in older adults, and this is speculated to be due to low blood fats being linked with markers for poorer health such as subclinical disease and frailty.^36^ Thus, it is possible that hypercholesterolemia is protective because low levels of cholesterol are linked with other risk factors for dementia. This could be examined in future work.

Previously, researchers have suggested that investigating cardiometabolic abnormalities as a whole might not offer any additive value in the prediction of dementia because of individual indicators of cardiometabolic abnormalities working in opposing directions.^37^, ^38^ However, this assumption is complicated by the fact that different studies will find opposing results when examining the same cardiometabolic abnormality. For instance, some studies have found that abdominal obesity,^11^ hypertension,^39^ and high lipids^11^, ^35^ are protective against risk of dementia. Other studies find that obesity,^40^, ^41^ hypertension,^42^ diabetes,^43^ and high lipids^44^ are risk factors for incident dementia. Finally, some studies find no association between any individual cardiometabolic risk factors with incident dementia.^10^ Therefore, there is a need for a lot more work to determine how cardiometabolic abnormalities as a whole and individually are linked with the risk of dementia and the effect of modifiers that may explain why these differences exist.

We found that the co‐occurrence of high depressive symptoms with either central obesity, hypertension, or hyperglycemia was linked with dementia in unadjusted analyses. However, these results were attenuated after adjustment for confounders. This suggests that the association between obesity, hypertension, and hyperglycaemia with dementia is explained by other factors, and the investigation of potential mediators in this relationship could tell us more about how cardiometabolic risk factors and depression are linked with dementia.

This study has several strengths including a large population‐based design, a long follow‐up period and objective blood measures for cardiometabolic risk factors. Moreover, the ELSA data set allowed for repeated assessment of dementia development every 2 years over the 10‐year follow‐up and evaluation of various potentially confounding risk factors.

Despite the strengths, this study also has several limitations that should be considered in the interpretation of the results. There is possibility of response bias because of self‐reported assessment of dementia incident and date of diagnosis without being validated by a clinical diagnosis.

Depressive symptoms were assessed using the CES‐D scale, which is considered a screening tool rather than a clinical diagnostic tool. As well, the CES‐D scale assesses self‐reported depressive symptoms experienced within the past week and does not account for history and treatment of depression. Another limitation may lie in the fact that the thresholds used for defining cardiometabolic abnormalities have not been standardised in older individuals and, thus, might not capture in the elderly what they intend to capture in middle age because of metabolic changes with ageing.^34^

Moreover, the lack of data on VaD within ELSA could be problematic. ELSA does have information on AD, however all other dementias are collapsed into a single category. Given that previous work suggests that cardiometabolic abnormalities are predictive of VaD rather than other dementias,^32^, ^33^ this could mean that we did not find significant results as we were unable to investigate this subtype of dementia.

Survival bias is also a perennial concern in longitudinal studies examining older individuals. It is possible that the oldest groups with cardiometabolic abnormalities died before developing dementia, and the sample may be representative of healthy survivors who are less susceptible to dementia caused by cardiometabolic risk factors.^11^ There is also the possibility that many cases of dementia may have been censored as people developing this disorder could have been more likely to drop out. Thus, there is a possibility of underestimation of the association between depression, cardiometabolic abnormalities, and dementia incidence.

Future research is needed to elucidate the association between cardiometabolic abnormalities and risk of dementia, examining the interactions between single cardiometabolic risk factors and dementia separately, as well as to extend the analyses to specific etiologic subtypes of dementia (VaD and AD). Further studies could also investigate the role of other inflammatory markers (eg, Interleukin‐6) in the development of dementia.

Our findings show that depression is an important risk factor for dementia. Given that depression is potentially modifiable, future studies are needed to determine whether effective depression interventions in later life have any effect in reducing the future risk of dementia. Clinicians should be vigilant for late‐life depression, which may represent a manifestation of the incipient development of dementia, and thus, carefully follow up these older individuals for future cognitive impairment. Drawing attention to the role of late‐life depression in the risk of developing dementia, and identifying the underlying mechanisms between these two conditions will not only provide substantial insights into the causes of dementia but will also inspire novel strategies for preventing and treating dementia.

## 7 CONCLUSION

Overall, this study provides additional evidence for the link between depression and future risk of dementia, supporting that older individuals with high depressive symptoms are associated with an increased risk of subsequent dementia. However, cardiometabolic abnormalities both with and without co‐occurring depressive symptoms were not linked with incident dementia.

## CONFLICT OF INTEREST

None declared.

## AUTHORSHIP

Both authors designed the study with P.K. completing the study analyses. Both P.K. and K.S. drafted the first version of the manuscript and P.K. completed the revisions of the manuscript. Both authors give approval of the study to be published.

## FUNDING

There was no funding for this research.

## Supporting information

Supporting info item

Supporting info item

Supporting info item
