# Supplementary material for: Risk of dementia associated with cardiometabolic abnormalities and depressive symptoms: a longitudinal cohort study using the English longitudinal study of ageing
Source: Int J Geriatr Psychiatry. 2018 Nov 27;34(2):289–98. doi: 10.1002/gps.5019 (PMC6587526; doi:10.1002/gps.5019)
Supplement: Supplementary file 1 — Supporting info item [file GPS-34-289-s001.docx]

Table A

Supplementary Table of Sensitivity Analysis using Cox Proportional Hazards Regression across depressive symptoms and cardiometabolic abnormalities groups with 2-year

time lag for dementia diagnosis

| *Hazard Ratio (95% CI) of Dementia* | | | | |
| --- | --- | --- | --- | --- |
| Cox Regression HR (95% CI) | noDnoCM | DnoCM | noDCM | DCM |
| Model 1: Unadjusted | 1.00 | 2.57 ***  (1.54, 4.29) | 1.32  (0.94, 1.86) | 1.82*  (1.06, 3.16) |
| Model 2: Adjusted for age, gender, education, marital status and net wealth | 1.00 | 2.10 **  (1.24, 3.56) | 1.11  (0.79, 1.58) | 1.18  (0.68, 2.09) |
| Model 3: Model 2 + adjusted for cardiovascular comorbidity, smoking status and physical activity | 1.00 | 1.79 *  (1.05, 3.06) | 1.04  (0.73, 1.48) | 0.98  (0.55, 1.75) |
| Model 4: Model 3 + adjusted for cognitive function | 1.00 | 1.27  (0.74, 2.19) | 0.98  (0.69, 1.40) | 0.70  (0.39, 1.25) |
| *Note*. HR = hazard ratio. CI = confidence interval. noDnoCM: no or low depressive symptoms and no cardiometabolic abnormalities group; DnoCM: high depressive symptoms only group; noDCM: cardiometabolic abnormalities only group; DCM: comorbid high depressive symptoms and cardiometabolic abnormalities group.  * p<.05 ** p<.01 *** p<.001 | | | | |
