# Supplementary material for: Risk of dementia associated with cardiometabolic abnormalities and depressive symptoms: a longitudinal cohort study using the English longitudinal study of ageing
Source: Int J Geriatr Psychiatry. 2018 Nov 27;34(2):289–98. doi: 10.1002/gps.5019 (PMC6587526; doi:10.1002/gps.5019)
Supplement: Supplementary file 2 — Supporting info item [file GPS-34-289-s002.docx]

Table B

Supplementary Table of Sensitivity Analysis using Cox Proportional Hazards Regression for the cases without cardiovascular co-morbidity at baseline

| *Hazard Ratio (95% CI) of Dementia* | | | | |
| --- | --- | --- | --- | --- |
| Cox Regression HR (95% CI) | noDnoCM | DnoCM | noDCM | DCM |
| Model 1: Unadjusted | 1.00 | 2.17 *  (1.13, 4.17) | 1.48*  (1.01, 2.17) | 1.35  (0.64, 2.86) |
| Model 2: Adjusted for age, gender, education, marital status and net wealth | 1.00 | 2.06 *  (1.05, 4.02) | 1.31  (0.89, 1.94) | 1.08  (0.50, 2.30) |
| Model 3: Model 2 + adjusted for smoking status and physical activity | 1.00 | 1.86  (0.94, 3.68) | 1.28  (0.86, 1.89) | 0.95  (0.44, 2.05) |
| Model 4: Model 3 + adjusted for cognitive function | 1.00 | 1.19  (0.59, 2.38) | 1.17  (0.79, 1.73) | 0.57  (0.26, 1.26) |
| *Note*. HR = hazard ratio. CI = confidence interval. noDnoCM: no or low depressive symptoms and no cardiometabolic abnormalities group; DnoCM: high depressive symptoms only group; noDCM: cardiometabolic abnormalities only group; DCM: comorbid high depressive symptoms and cardiometabolic abnormalities group.  * p<.05 ** p<.01 *** p<.001 | | | | |
