# Supplementary material for: Risk of dementia associated with cardiometabolic abnormalities and depressive symptoms: a longitudinal cohort study using the English longitudinal study of ageing
Source: Int J Geriatr Psychiatry. 2018 Nov 27;34(2):289–98. doi: 10.1002/gps.5019 (PMC6587526; doi:10.1002/gps.5019)
Supplement: Supplementary file 3 — Supporting info item [file GPS-34-289-s003.docx]

Table C

Supplementary Table of Sensitivity Analysis using Cox Proportional Hazards Regression for the cases with self-reported physician diagnosed dementia excluding cases diagnosed via the IQCODE

| *Hazard Ratio (95% CI) of Dementia* | | | | |
| --- | --- | --- | --- | --- |
| Cox Regression HR (95% CI) | noDnoCM | DnoCM | noDCM | DCM |
| Model 1: Unadjusted | 1.00 | 2.14 **  (1.27, 3.58) | 1.29  (0.93, 1.79) | 1.52  (0.87, 2.66) |
| Model 2: Adjusted for age, gender, education, marital status and net wealth | 1.00 | 1.43  (0.84, 2.45) | 1.08  (0.77, 1.51) | 1.02  (0.58, 1.81) |
| Model 3: Model 2 + adjusted for cardiovascular comorbidity, smoking status and physical activity | 1.00 | 1.26  (0.73, 2.16) | 1.02  (0.73, 1.42) | 0.83  (0.47, 1.50) |
| Model 4: Model 3 + adjusted for cognitive function | 1.00 | 0.96  (0.55, 1.65) | 1.00  (0.72, 1.40) | 0.59  (0.33, 1.06) |
| *Note*. HR = hazard ratio. CI = confidence interval. noDnoCM: no or low depressive symptoms and no cardiometabolic abnormalities group; DnoCM: high depressive symptoms only group; noDCM: cardiometabolic abnormalities only group; DCM: comorbid high depressive symptoms and cardiometabolic abnormalities group.  * p<.05 ** p<.01 *** p<.001 | | | | |
